# Quantitative Electroencephalography Markers for an Accurate Diagnosis of Frontotemporal Dementia: A Spectral Power Ratio Approach

**DOI:** 10.3390/medicina59122155

**Published:** 2023-12-13

**Authors:** Jinwon Chang, Chul Chang

**Affiliations:** 1 Korean Minjok Leadership Academy, Hoengseong 25268, Republic of Korea; 2 College of Medicine, Catholic University of Korea, Seoul 06591, Republic of Korea; janita7@naver.com

**Keywords:** electroencephalography, power ratio, frontal lobe, temporal lobe, dementia

## Abstract

*Background and Objectives*: Frontotemporal dementia (FTD) is the second most common form of presenile dementia; however, its diagnosis has been poorly investigated. Previous attempts to diagnose FTD using quantitative electroencephalography (qEEG) have yielded inconsistent results in both spectral and functional connectivity analyses. This study aimed to introduce an accurate qEEG marker that could be used to diagnose FTD and other neurological abnormalities. *Materials and Methods*: We used open-access electroencephalography data from OpenNeuro to investigate the power ratio between the frontal and temporal lobes in the resting state of 23 patients with FTD and 29 healthy controls. Spectral data were extracted using a fast Fourier transform in the delta (0.5 ≤ 4 Hz), theta (4 ≤ 8 Hz), alpha (8–13 Hz), beta (>13–30 Hz), and gamma (>30–45 Hz) bands. *Results*: We found that the spectral power ratio between the frontal and temporal lobes is a promising qEEG marker of FTD. Frontal (F)-theta/temporal (T)-alpha, F-alpha/T-theta, F-theta/F-alpha, and T-beta/T-gamma showed a consistently high discrimination score for the diagnosis of FTD for different parameters and referencing methods. *Conclusions*: The study findings can serve as reference for future research focused on diagnosing FTD and other neurological anomalies.

## 1. Introduction

Frontotemporal dementia (FTD) is the second most common presenile dementia, with an annual prevalence and incidence of 0.01–4.6 and 0.0–0.3 per 1000 people, respectively [1]. However, because FTD is less frequent than Alzheimer’s disease [2], FTD has been relatively poorly investigated. Moreover, the psychiatric symptoms associated with FTD, such as interpersonal conduct and personal regulation, sometimes lead to misdiagnoses of depression or bipolar disorder [3]. Although brain imaging methods, including magnetic resonance imaging, can identify variants of frontotemporal dementia, they are expensive and restricted to early-stage diagnosis. Hence, further investigations of inexpensive and objective markers of FTD are required.

Quantitative electroencephalography (qEEG) is an inexpensive, noninvasive, and convenient tool for assessing alterations in neuronal activity after dementia. qEEG enables the objective evaluation of neurological abnormalities in cognitive function by manifesting the degree of synchronization or desynchronization of connected neurons. The functional connectivity (FC) and spectral power of electroencephalography (EEG) are mainly used to distinguish patients with different types of dementia from healthy controls (CTLs) [4]. However, with respect to FTD, qEEG studies attempting to distinguish FTD from healthy controls have hitherto been unsuccessful. First, spectral power approaches have yielded contradictory results. Lower global-averaged alpha, beta, and spectral ratios between slow and fast rhythms were observed in patients with FTD compared with those in normal CTLs [5]. However, another study using similar global field power analyses identified only a decreased alpha in FTD [6]. 

In regional analyses, no significance was found in alpha and spectral ratios, whereas increased frontal theta and delta power, increased parietal theta, and decreased temporal and parietal beta power were observed in patients with FTD compared with those in healthy participants used as CTLs [7]. In contrast, using the same regional analyses, no significant changes were detected in these indicators for Pick’s disease, a variant of FTD [8]. Using standardized low-resolution electromagnetic tomography (sLORETA) revealed only lower frontal and temporal alpha power in patients with FTD compared with those in normal CTLs [6]. However, using the same sLORETA and spectral power methods, another study observed increased theta power over all regional lobes in FTD [9]. In particular, for spectral power approaches, despite the analyses using the same method (e.g., sLORETA, global field power, and spectral power of regional electrodes), each study resulted in a different significance in different frequency bands in different lobes. Such an inconsistency may result from the inappropriateness of previous spectral power indices in accurately capturing abnormalities in FTD. 

Despite their excellent ability to obtain phase information combined with EEG amplitudes, FC analyses are highly inconsistent. Several EEG FC studies have failed to produce consistent results regarding depression [10]. Such inconsistencies in FC were also observed in empirical studies on FTD. Synchronization likelihood, mean clustering coefficient (local connectivity), characteristic path length (global connectivity), and global minimum spanning tree measures analyses found no difference in FC between patients with FTD and CTLs [11,12,13]. However, an increase in the degree of correlation in the lower alpha band was observed in patients with FTD [12]. In contrast, no significant difference in the alpha and beta bands in the phase lag index was observed between patients with FTD and CTLs; instead, an increased phase lag index in the delta band was observed [13]. In addition, hypoconnectivity in the mid- and long-range frontotemporal networks in the alpha and beta bands was observed via weighted symbolic mutual information in the behavioral variant of FTD [14]. Determining whether these opposing evaluations of FTD result from state dependency, methodological problems, or the use of slightly different indexes is difficult, exposing scientists to the possibility of interpretational pitfalls unless advanced statistical evaluation or meta-analysis is conducted [15]. Additionally, FC is sometimes a weak parameter for determining causal brain interactions [16]. These studies indicated that FC may not be appropriate for diagnosing FTD.

Therefore, new EEG markers, as an alternative to the previously inconsistent qEEG methods, should be examined for distinguishing patients with FTD from healthy CTLs. This new qEEG index should be related to the neurological symptoms and pathology of FTD because patients with FTD show EEG and neuropathological patterns that are distinctive from those of patients with Parkinson’s or Alzheimer’s disease [17,18,19,20,21]. The association between the frontal and temporal lobes appears to be a plausible and reasonable indicator for the diagnosis of FTD, as severe frontotemporal degeneration is a distinctive pathological hallmark of FTD. Impaired interactions in the frontotemporal network in patients with FTD represent different interactions between the frequency bands of each lobe [21,22]. Therefore, the relationships between the temporal and frontal frequency bands may be promising indicators of FTD. 

In addition, the spectral ratios between frequency bands can be used to successfully evaluate cognition and detect dementia, including Alzheimer’s disease, Parkinson’s disease, and Lewy body dementia [23,24,25,26,27,28]. However, the EEG spectral ratios of frontal frequency power/temporal frequency power have not yet been investigated. The current study aimed to introduce a new, accurate, and consistent qEEG marker that can be used to diagnose FTD and other neurological abnormalities. We hypothesized that the spectral power ratio between the frontal and temporal lobes would consistently and successfully detect FTD. The study focused on the spectral power ratio between the frontal and temporal lobes as an efficient and effective qEEG marker for detecting FTD. The effects of different parameters in the measurement of spectral power were compared to determine the consistency of this index.

## 2. Materials and Methods

### 2.1. Participants

The Statistical Package for the Social Sciences version 25.0 (IBM Corp., Armonk, NY, USA) and MedCalc^®^ Statistical Software version 20.218 (MedCalc Software Ltd., Ostend, Belgium); https://www.medcalc.org (accessed on 5 December 2023) were used for all statistical analyses. We calculated sample sizes for area under ROC curve using MedCalc. We observed that 20 samples for both negative and positive cases are enough for a type 1 error of 0.01, a type 2 error of 0.2, an area under ROC curve of 0.8, and a null hypothesis value of 0.5, with disease prevalence fixed at 50% (what we used for PPV and NPV in the analysis). Preprocessed datasets from OpenNeuro were used [29,30,31]. In total, 23 patients with FTD and 29 healthy CTLs at the Second Department of Neurology, AHEPA General Hospital, Thessaloniki, Greece, were included in the dataset. The initial diagnosis of patients with FTD was performed according to the criteria provided by the Diagnostic and Statistical Manual of Mental Disorders, 3rd ed., revised (DSM-IIIR, DSM IV, ICD-10), and the National Institute of Neurological, Communicative Disorders and Stroke—Alzheimer’s Disease and Related Disorders Association (NINCDS—ADRDA) [29,30,31]. For patients with FTD, no dementia-related comorbidities, epilepsy, cortical brain lesions, or electrolyte disorders were reported. The cognitive state of each participant was assessed using the International Mini-Mental State Examination (scale: 0–30). Lower Mini-Mental State Examination scores indicated cognitive impairment. The disease duration was measured in months (first quartile, 24 months; second quartile, 25 months; third quartile, 28.5 months). 

The original study In OpenNeuro was approved by the Scientific and Ethics Committee of AHEPA University Hospital, Aristotle University of Thessaloniki, under protocol number 142/12-04-2023. This study was approved by the Public Institutional Review Board Designated by the Ministry of Health and Welfare (P01-202304-01-023) and was conducted according to the tenets of the Declaration of Helsinki. All participants provided written informed consent.

### 2.2. EEG Acquisition and Preprocessing

Each participant was subjected to an eye-closed resting-state EEG at the Second Department of Neurology of the AHEPA General Hospital of Thessaloniki, Greece. A Nihon Kohden EEG 2100 device (Tokyo, Japan) with a 10/20 system and sampling frequency of 500 Hz was used. The two electrodes on the mastoids were used as references. Each recording lasted over 10 min. Each EEG dataset was preprocessed from an original study [31]. A 0.5–45 Hz filter was applied. After being re-referenced to A1–A2, the raw data were cleaned with the ASR plugin in EEGLAB [32,33]. An independent component analysis was conducted to distinguish and remove artifactual brain components in the eyes and muscles. Finally, all data were average-referenced.

### 2.3. Spectral Analysis

The absolute average spectral power of each frequency band of delta (0.5 ≤ 4 Hz), theta (4 ≤ 8 Hz), alpha (8–13 Hz), beta (>13–30 Hz), and gamma (>30–45 Hz) was calculated using the discrete fast Fourier transform (FFT) at a 2-s FFT window length and 10 steps per Hz. The FFT algorithm mathematically transformed EEG signals from the time domain into the frequency domain to obtain the spectra by decomposing signals into sinusoidal components at all frequencies. The spectral power was separated into frontal (electrodes Fp1, Fp2, F3, F4, F7, F8, and Fz) and temporal (electrodes T3, T4, T5, and T6) power. The frontal and temporal power in each frequency band were calculated, and inter-lobar and intra-lobar power ratios for every combination of each frequency band were calculated (frontal frequency power/temporal frequency power, frontal frequency power/frontal frequency power, or temporal frequency power/temporal frequency power). For the consistency test, 2 s/5 and 1 s/10 steps were additionally applied for the FFT window length/step size per Hz to measure the same spectral power. The 2 s/10 step (FFT window length/step size per Hz) represented 1000/5000/500 samples (window/FFT/overlapping length), whereas the 2 s/5 step represented 1000/2500/500 samples. The 1 s/10 step represented 500/5000/250 samples. In addition, because average reference might be invalid for low-density electrodes, infinity reference (EEGLAB plugin REST) was applied to compare results at 2 s/10 s [34,35].

### 2.4. Statistical Analysis

The clinicodemographic patient characteristics were assessed using rank-sum and chi-square tests as data were nonnormally distributed. The Mann–Whitney U test was used to determine the difference in the frontal/temporal brain ratio between patients with FTD and CTL. The Benjamini–Hochberg procedure was applied to correct the false discovery rate. Only variables significantly different between the two groups were included in statistical analyses. Receiver operating characteristic (ROC) and precision-recall (PR) curve analyses were performed to determine the diagnostic accuracy of each spectral power ratio in the frontal and temporal lobes. The area under the curve (AUC) of the ROC and PR was compared using Delong’s method and the logit method, respectively [36,37,38]. With a standardized prevalence of 50%, Youden’s index was used to determine specificity, sensitivity, cutoff point, positive predictive value, and negative predictive value [39,40,41]. Bootstrapping with 5000 iterations and 978 seeds was applied to measure the confidence interval of the maximum Youden’s index and the optimal cutoff point. Multiple logistic regression with a stepwise method (enter variables if probability < 0.05 and remove if >0.1) was applied with covariates of age and sex to determine the discrimination ability of lobar spectral power and F/T spectral power ratios. The same statistical analyses were applied to the spectral power using different FFT parameters (2 s/5 s and 1 s/10 s). 

## 3. Results

**Figure 1:**
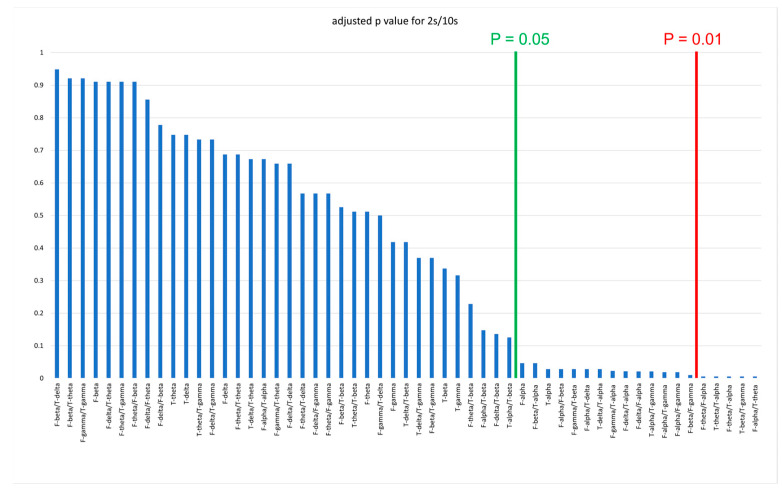
Differences in spectral power ratio between FTD and CTL (expressed as adjusted p value). The *p* value was adjusted after FDR correction using the Benjamini–Hochberg procedure at 2 s/10 step for FFT window length/step size per Hz. The green and red lines represent criteria of *p* < 0.05 and *p* < 0.01, respectively. FTD: frontotemporal dementia; CTL: control; FDR: false discovery rate; F: frontal; T: temporal.

**Figure 2:**
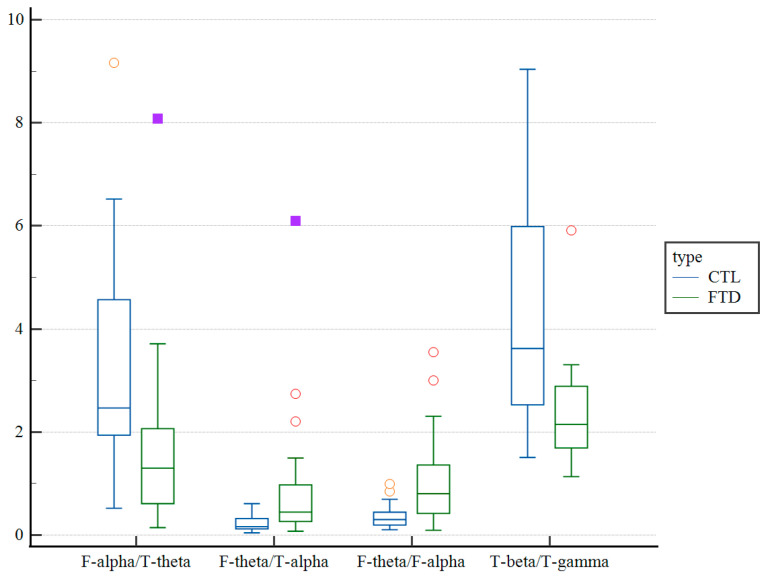
Distribution plot of each of the four spectral power ratios at 2 s/10 s with average referencing. A box-and-whisker plot is applied to present a statistical summary of each variable between the FTD and CTL groups. The blue and green boxes represent the CTL and FTD groups, respectively. The central box represents the values from the lower to the upper quartile (25 to 75 percentile). The middle line represents the median. The horizontal line extends from the minimum to the maximum value, excluding outside and far-out values which are displayed as separate points. The yellow and red hollow circle dots represent an outside value defined as a value that is smaller than the lower quartile minus 1.5 times the interquartile range, or larger than the upper quartile plus 1.5 times the interquartile range (inner fences). The purple solid rectangle dots represent a far-out value defined as a value that is smaller than the lower quartile minus 3 times the interquartile range, or larger than the upper quartile plus 3 times the interquartile range (outer fences). FTD: frontotemporal dementia; CTL: control; F: frontal; T: temporal.

We did not detect any significant differences in age (*p* = 0.068) and sex (*p* = 0.930) between groups. However, the FTD group showed lower Mini-Mental State Examination scores (*p* < 0.001) compared with the CTL group. The clinicodemographic characteristics of the patients are presented in Table 1. For a condensed and effective comparison, we performed the Mann–Whitney U test to select consistently significant indices of the spectral power ratio between the frontal and temporal lobes (Figure 1). Regarding individual regional power, we observed that the FTD group showed significantly smaller frontal and temporal alpha powers than those in the CTL group (*p* < 0.05), whereas in the case of the spectral power ratio between the frontal and temporal lobes, the FTD group showed larger frontal (F)-delta/temporal (T)-alpha (*p* < 0.05), F-theta/T-alpha (*p* < 0.01), F-beta/T-alpha (*p* < 0.05), F-gamma/T-alpha (*p* < 0.05), F-gamma/T-beta (*p* < 0.05), F-delta/F-alpha (*p* < 0.05), F-theta/F-alpha (*p* < 0.01), T-delta/T-alpha (*p* < 0.05), and T-theta/T-alpha (*p* < 0.01) ratios compared with those in the CTL group. We observed smaller spectral power ratios for F-alpha/T-delta (*p* < 0.05), F-alpha/T-theta (*p* < 0.01), F-alpha/T-gamma (*p* < 0.05), F-alpha/F-beta (*p* < 0.05), F-alpha/F-gamma (*p* < 0.05), F-beta/F-gamma (*p* < 0.05), T-alpha/T-gamma (*p* < 0.05), and T-beta/T-gamma (*p* < 0.01) during FTD. The significance tests for each FFT parameter and referencing method are presented in Figure 1 and Supplementary Figures S1–S3. We found that with different referencing methods and different FFT parameters, T-alpha, F-delta/T-alpha, F-theta/T-alpha, F-alpha/T-theta, F-alpha/T-gamma, F-gamma/T-alpha, F-gamma/T-beta, F-delta/F-alpha, F-theta/F-alpha, F-alpha/F-beta, F-alpha/F-gamma, F-beta/F-gamma, T-theta/T-alpha, T-alpha/T-gamma, and T-beta/T-gamma consistently showed statistically significant differences between the FTD and CTL groups (*p* < 0.05). Among these statistically significant indices, we selected robust and consistent variables with the significance criterion of *p* < 0.01 (F-theta/T-alpha, F-alpha/T-theta, F-theta/F-alpha, and T-beta/T-gamma) for further analysis. The distribution of each of the four spectral power ratios for each FFT parameter and referencing method is presented in Figure 2 and Supplementary Figures S4–S6. Only F-theta/T-alpha and F-theta/F-alpha were consistently higher in the FTD group than in the CTL group (Figure 2). Meanwhile, we noticed that the F-alpha/T-theta and T-beta/T-gamma were consistently lower in the FTD group compared with those in the CTL group (Figure 2). 

**Figure 3:**
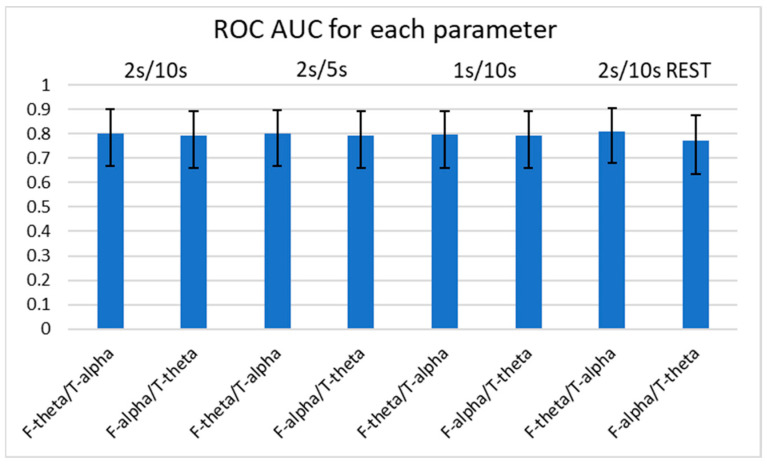
AUCs of ROC curves of F-theta/T-alpha and F-alpha/T-theta for each parameter. Error bars represent the 95% confidence intervals. No significant differences in AUCs between spectral power ratios or between parameters was found using Delong’s method. AUC: area under curve; ROC: receiver operating characteristic.

**Figure 4:**
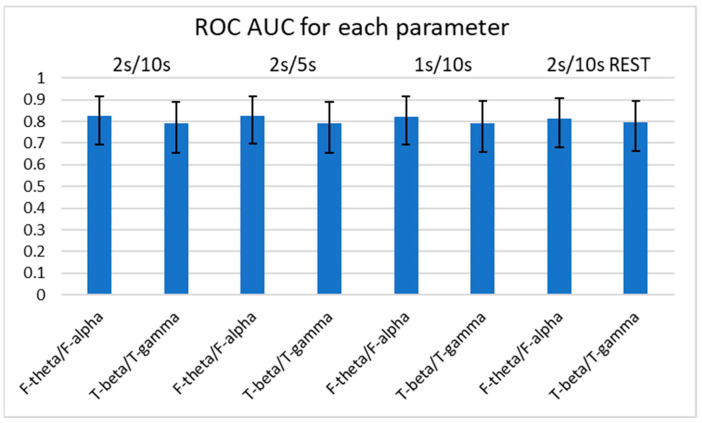
AUCs of ROC curves of F-theta/F-alpha and T-beta/T-gamma for each parameter. Error bars represent the 95% confidence intervals. No significant differences in AUCs between spectral power ratios or between parameters was found using Delong’s method. AUC: area under curve; ROC: receiver operating characteristic.

Using Delong’s method for diagnostic sensitivity and specificity, we calculated the AUC of ROC curves among the four consistently significant spectral power ratios with *p* < 0.01 for determining the occurrence of FTD (Figure 3 and Figure 4). Of note, we examined the spectral power ratios that showed significant differences between the FTD and CTL groups for consistency. We determined that the AUCs of all indices (F-theta/T-alpha, F-alpha/T-theta, F-theta/F-alpha, and T-beta/T-gamma) were over 0.7. The F-theta/F-alpha power had the highest AUC score compared with the other power ratios for every parameter. However, we did not observe any significant difference in the AUC among the indices. The F-theta/F-alpha had the highest discrimination score at 2 s/5 s (AUC: 0.826 ± 0.111), which was only 1% greater than its lowest score at 2 s/10 s with REST (AUC: 0.814 ± 0.113). The F-alpha/T-theta had the highest discrimination score at 2 s/10 s, 2 s/5 s, and 1 s/10 s (AUC: 0.793 ± 0.133), which was only 3% greater than its lowest score at 2 s/10 s with REST (AUC: 0.772 ± 0.121). Interestingly, we found that neither varying parameters nor referencing methods changed the AUCs of the four power ratios. We did not observe any significant differences in AUCs between different parameters either.

**Figure 5:**
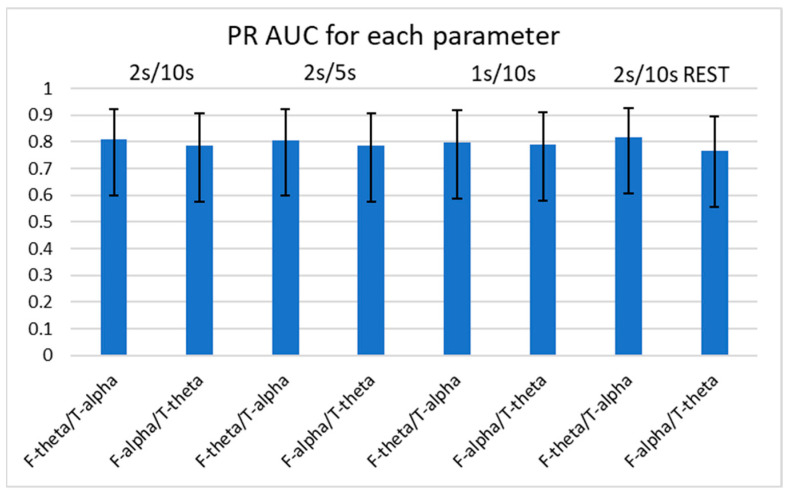
AUCs of PR curves of F-theta/T-alpha and F-alpha/T-theta for each parameter. Error bars represent 95% confidence intervals. No significant difference in AUC between spectral power ratios or between parameters was found using the logit method. AUC: area under curve; PR: precision recall.

**Figure 6:**
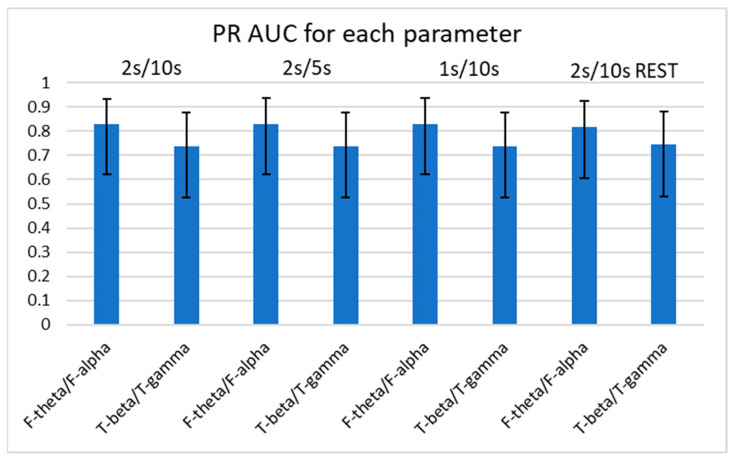
AUCs of PR curves of F-theta/F-alpha and T-beta/T-gamma for each parameter. Error bars represent 95% confidence intervals. No significant difference in AUC between spectral power ratios or between parameters was found using the logit method. AUC: area under curve; PR: precision recall.

Using the logit and nonlinear interpolation method, we calculated the AUC of PR curves of the four consistently significant spectral power ratios for diagnostic sensitivity and positive predictive value in the determination of the presence of FTD (Figure 5 and Figure 6). We further examined the spectral power ratios that showed significant differences between the FTD and CTL groups for consistency. We found that the F-theta/F-alpha power had the highest AUC (0.831 ± 0.157) at 2 s/5 s, which was only 2% greater than its lowest score at 2 s/10 s with REST (AUC: 0.816 ± 0.160). The F-alpha/T-theta power had the highest AUC (0.791 ± 0.166) at 1 s/10 s, which was 3% greater than its lowest score at 2 s/10 s with REST (AUC: 0.767 ± 0.170). Similarly, we determined that neither varying parameters nor referencing methods changed the AUCs of the four power ratios. However, we did not observe any significant differences in the AUCs between different parameters or among the four spectral power ratios.

The indicators of diagnostic accuracy, including sensitivity, specificity, positive predictive value (PPV), and negative predictive value (NPV) at the optimal cutoff point using Youden’s index, are presented in Table 2 and Table 3. For standardization, we used a prevalence of 50% to estimate the predictive values. At 2 s/10 s, we did not detect any significant difference in the cutoff point, sensitivity, specificity, maximum Youden’s index, PPV, and NPV between the two spectral power ratios, as indicated by an overlapping 95% confidence interval. The diagnostic accuracy values for each parameter at the optimal cutoff point by Youden’s index are also presented in Table 2 and Table 3. We further examined the spectral power ratios that showed significant differences between the FTD and CTL groups for consistency. For every FFT and referencing method, no significant differences were observed in the cutoff point, sensitivity, specificity, maximum Youden’s index, PPV, and NPV among the spectral power ratios, as indicated by an overlapping 95% confidence interval. 

We applied a multiple logistic regression model with stepwise variable selection to determine whether fluctuations in each spectral power ratio led to a higher probability of developing FTD (Table 4). We found that at 2 s/10 s, only the F-theta/F-alpha power ratio was recognized as a significant independent variable affecting the probability of FTD. In the model, an increase in the F-theta/T-alpha power ratio significantly increased the likelihood of developing FTD (odds ratio = 51.782, 95% CI: 4.287–625.529). The results of multiple logistic regression analysis using stepwise variable selection for each parameter are shown in Table 4 for consistency. We observed that at 2 s/10 s, 2 s/5 s, 1 s/10 s, and 2 s/10 s with REST, the F-theta/F-alpha power ratio was consistently a significant independent variable affecting the probability of developing FTD. In all models, an increase in the F/T theta/alpha power ratio significantly increased the likelihood of FTD (odds ratio > 1). 

## 4. Discussion

Previous attempts to diagnose FTD using qEEG have reported conflicting findings. In this study, we found that spectral power ratios between the frontal and temporal lobes have more accurate and consistent diagnostic power for detecting FTD than individual regional power in a certain lobe (e.g., frontal alpha and temporal alpha). For every FFT parameter and referencing method used in the current study, F-theta/T-alpha, F-alpha/T-theta, F-theta/F-alpha, and T-beta/T-gamma were consistently altered in the FTD group. No such consistency was observed when the individual regional powers of the FTD and CTL groups were compared. These F/T power ratios also consistently showed a mean AUC around 0.8, indicating very good accuracy [42]. Moreover, the AUCs of the ROC and PR curves were maintained, although the FFT parameters were modified. 

The sensitivity, specificity, Youden’s index, PPV, and NPV at the optimal cutoff point from the maximum Youden’s index were also maintained despite the variations in FFT parameters and referencing methods. This result implied that compared with individual frequency power in a certain lobe (e.g., frontal alpha, temporal theta, and temporal alpha), the spectral power ratio between specific frequency bands is more consistent in distinguishing between patients with FTD and CTLs, regardless of which parameters are used for signal processing. In particular, although different FFT parameters usually lead to different comparison results owing to their effects on the trade-off between time and frequency resolution, some spectral ratios are likely to be resistant to such parameter fluctuations, at least for FTD. This further supported their high usefulness and validity for the diagnosis of FTD. Importantly, this simple approach can lay the foundation for the implementation of several easier applications for FTD diagnosis. Combining the results from analyses using different FFT parameters and referencing methods, the current study demonstrated that F-theta/T-alpha, F-alpha/T-theta, F-theta/F-alpha, and T-beta/T-gamma exhibited consistently high AUCs, showing a consistently high causal association with the likelihood of developing FTD. Hence, among spectral power ratios between the frontal and temporal lobes, F-theta/T-alpha, F-alpha/T-theta, F-theta/F-alpha, and T-beta/T-gamma could be the most effective diagnostic markers for FTD.

Patients with FTD experience severe frontotemporal degeneration. However, this does not imply that all frequency bands oscillating in the frontotemporal region are impaired. Each cross frequency interaction is a unique representative of a distinctive neuronal network in which different frequency bands represent different groups of synchronized or desynchronized neurons [22]. The current study used average spectral power to express distinctive neuronal networks that are altered by frontotemporal degeneration as a relationship between certain frontal frequency powers and certain temporal frequency powers. Alterations in F-theta/T-alpha, F-alpha/T-theta, and F-theta/F-alpha may indicate impairments in the network relating theta and alpha oscillations due to frontotemporal degeneration, which could be related to changes in the temporal, frontal, and subcortical functional network in the insula, anterior cingulate cortex, temporal lobe, medial frontal lobe, and orbitofrontal cortex [21,43,44,45,46]. For example, a dramatic increase in F-theta/T-alpha may be attributed to both the increase in frontal theta and decrease in temporal alpha. An increased frontal theta may also be associated with the frontal midline theta rhythm that is negatively associated with sympathetic activation, which is frequently impaired, resulting in bradycardia in FTD, unlike in Alzheimer’s disease and other types of dementia [47,48]. A decreased temporal alpha may be affected by abnormal thalamic activity and related reduced glucose metabolism in FTD [49,50,51,52,53]. Meanwhile, alterations in the temporal gamma rhythm in T-beta/T-gamma may imply typical impairments of executive functions and the hippocampus in patients with FTD, both of which are highly associated with gamma oscillations [54,55,56]. Alterations in the temporal beta rhythm in T-beta/T-gamma may be associated with the impaired ability to predict others’ behaviors in FTD [57,58]. However, interpreting the differences in spectral power ratios remains a challenge. First, precisely associating certain frequency powers with specific regions of the brain is difficult. Further simultaneous EEG and functional magnetic resonance imaging analyses or LORETA are likely to solve these spatial resolution problems. Second, the lack of phase information in spectral power ratios indicates that spectral power ratios may represent simultaneous alterations in various neuronal networks [22]. This time-ignored feature makes it impossible to associate spectral power ratios with specific functional networks (e.g., the default mode network) because many neuronal networks and their consequences are mixed and inseparable. However, compared with functional connectivity and other phase-coupled analyses, space-based connectivity is less susceptible to state dependency, possibly leading to more consistent and distinguishable differences between patients with FTD and healthy CTLs [10,59]. Third, a paucity of sample sizes and variability in FFT parameters might have led to an over- or underestimation of the results of the current study. The three different variations of the FFT parameters used here may be insufficient for testing the consistency of these analyses. 

Fourth, although the diagnostic accuracy was not altered after the FFT parameters were modified, slight differences (not statistically different) were detected in the AUCs of ROC and PR curves between average and infinity reference methods. Because different referencing methods have different advantages and limitations, further analyses are needed for determining their clinical usefulness. Fifth, further investigations are needed to distinguish the spectral power ratios of FTD from those of Alzheimer’s and other dementias. Neurologically distinctive abnormalities in FTD might hint at the means by which we can determine whether altered inter-lobar and intra-lobar spectral power ratios are essentially overlapping features among dementias or caused by different sources [60]. Sixth, representative accuracy indicators, such as sensitivity, PPV, and AUC of the ROC curve, do not represent diagnostic accuracy in real tests. The optimal cut-off points in the current study were determined using Youden’s index. However, the Youden’s index method assumes equal weights on sensitivity and specificity, ignores the costs of decisions, and pursues only the maximized sum of sensitivity and specificity, which clearly is not the case in the real world. In addition, disease prevalence was standardized at 50% to estimate the PPV and NPV, whereas the actual prevalence was much lower. Nevertheless, these representative values could be used for a simpler and easier comparison with other diagnostic markers because Youden’s index provides a general overview of diagnostic accuracy, and the actual disease prevalence, or pretest probability, is usually not fixed. Several variants of FTD, different disease durations, and different cognitive states among patients with FTD may affect the spectral power ratios. Seventh, FTD subtypes such as behavioral variant frontotemporal dementia, semantic variants of primary progressive aphasia (PPA), and non-fluent variants of PPA are not stated in the current study. Because each subtype has different topographic characteristics, further analyses are needed to determine the clinical usefulness of spectral power ratios. However, structural brain changes and many clinical features such as cognitive-behavioral profiles are overlapping and dimensional (lying along a continuum) among different FTD subtypes [61,62,63]. Therefore, our study still has implications for a better understanding of FTD. Finally, our definition of frequency band is not general. We used delta (0.5 ≤ 4 Hz), theta (4 ≤ 8 Hz), alpha (8–13 Hz), beta (>13–30 Hz), and gamma (>30–45 Hz) for spectral analysis; however, the generally proposed ranges of delta (0.1 ≤ 4 Hz) and gamma (>30–80 Hz) are different [64]. Because we applied a high-pass filtering of 0.5 Hz and a low-pass filtering of 45 Hz to conduct independent component analysis with good decomposition and to remove noise such as channel and line noise, we adjusted the ranges of delta and gamma for a better extraction of spectral power [65]. 

## 5. Conclusions

In conclusion, despite the remaining challenges, the spectral power ratios between two lobes may be worth investigating further as a new qEEG parameter for screening and diagnosing FTD or possibly other dementias, such as Alzheimer’s and Parkinson’s diseases. In particular, diseases related to specific lobes may have distinctive spectral ratios between the frequency bands of abnormal lobes. The current study provided important baseline evidence for future studies on the EEG diagnosis of neurological abnormalities.

## Figures and Tables

**Table 1:** Comparison of clinicodemographic characteristics between patients with FTD and healthy controls.

	FTD Group	CTL Group	Significance
MMSE	22.17 (8.22)	30 (0)	*p* < 0.001
Age	63.6 (8.2)	67.9 (5.4)	*p* = 0.068
Sex	0.39	0.38	*p* = 0.930

MMSE and age are expressed as the mean ± standard deviation. Sex is expressed as the proportion of female participants. The Mann–Whitney U test and chi square test were used for comparison. A *p* value of <0.05 indicates statistical significance. FTD: frontotemporal dementia; CTL: control.

**Table 2:** Diagnostic accuracy of F-theta/T-alpha and F-alpha/T-theta for each parameter.

	2 s/10 s		2 s/5 s		1 s/10 s		2 s/10 s REST	
	F-Theta/T-Alpha	F-Alpha/T-Theta	F-Theta/T-Alpha	F-Alpha/T-Theta	F-Theta/T-Alpha	F-Alpha/T-Theta	F-Theta/T-Alpha	F-Alpha/T-Theta
Cutoff	>0.35 (0.16–0.61)	≤1.60 (1.44–5.12)	>0.35 (0.16–0.61)	≤1.60 (1.44–5.12)	>0.37 (0.18–0.63)	≤1.43 (0.77–1.79)	>0.24 (>0.12–0.36)	≤1.48 (1.30–4.92)
Sensitivity (%)	69.6 (47.1–86.8)	69.6 (47.1–86.8)	69.6 (47.1–86.8)	69.6 (47.1–86.8)	69.6 (47.1–86.8)	65.2 (42.7–83.6	78.3 (56.3–92.5)	73.9 (51.6–89.8)
Specificity (%)	79.3 (60.3–92.0)	86.2 (68.3–96.1)	79.3 (60.3–92.0)	86.2 (68.3–96.1)	79.3 (60.3–92.0)	89.7 (72.6–97.8)	72.4 (52.8–87.3)	75.9 (56.5–89.7)
Youden’s Index	0.489 (0.260–0.619)	0.558 (0.305–0.739)	0.489 (0.255–0.619)	0.558 (0.305–0.739)	0.489 (0.250–0.636)	0.549 (0.286–0.706)	0.507 (0.261–0.636)	0.498 (0.224–0.670)
PPV (%)	77.1 (61.1–87.8)	83.5 (66.1–92.9)	77.1 (61.1–87.8)	83.5 (66.1–92.9)	77.1 (61.1–87.8)	86.3 (67.5–95.0)	73.9 (60.2–84.2)	75.4 (60.6–85.9)
NPV (%)	72.3 (57.8–83.2)	73.9 (60.0–84.2)	72.3 (57.8–83.2)	73.9 (60.0–84.2)	72.3 (57.8–83.2)	72 (59.2–82.1)	76.9 (59.8–88.2)	74.4 (58.7–85.6)

Youden’s index is used to determine the optimal cutoff point with specific values for each index. The prevalence was standardized at 50%. The cutoff point, sensitivity, specificity, PPV, and NPV are expressed as the mean ± 95% confidence interval. PPV: positive predictive value; NPV: negative predictive value; F: frontal; T: temporal.

**Table 3:** Diagnostic accuracy of F-theta/F-alpha and T-beta/T-gamma for each parameter.

	2 s/10 s		2 s/5 s		1 s/10 s		2 s/10 s REST	
	F-Theta/F-Alpha	T-Beta/T-Gamma	F-Theta/F-Alpha	T-Beta/T-Gamma	F-Theta/F-Alpha	T-Beta/T-Gamma	F-Theta/F-Alpha	T-Beta/T-Gamma
Cutoff	>0.56 (0.25–0.70)	≤3.31 (2.98–5.91)	>0.56 (0.25–0.70)	≤3.31 (2.98–5.91)	>0.60 (0.27–0.86)	≤3.34 (3.11–5.96)	>0.65 (0.37–1.02)	≤3.30 (2.91–5.71)
Sensitivity (%)	69.6 (47.1–86.8)	95.7 (78.1–99.9)	69.6 (47.1–86.8)	95.7 (78.1–99.9)	69.6 (47.1–86.8)	95.7 (78.1–99.9)	65.2 (42.7–83.6)	95.7 (78.1–99.9)
Specificity (%)	86.2 (68.3–96.1)	55.2 (35.7–73.6)	86.2 (68.3–96.1)	55.2 (35.7–73.6)	86.2 (68.3–96.1)	55.2 (35.7–73.6)	86.2 (68.3–96.1)	55.2 (35.7–73.6)
Youden’s Index	0.558 (0.298–0.705)	0.508 (0.258–0.655)	0.558 (0.298–0.697)	0.508 (0.258–0.655)	0.558 (0.298–0.705)	0.508 (0.233–0.646)	0.514 (0.264–0.636)	0.508 (0.244–0.652)
PPV (%)	83.5 (66.1–92.9)	68.1 (58.5–76.3)	83.5 (66.1–92.9)	68.1 (58.5–76.3)	83.5 (66.1–92.9)	68.1 (58.5–76.3)	82.5 (64.5–92.5)	68.1 (58.5–76.3)
NPV (%)	73.9 (60.0–84.2)	92.7 (64.5–98.9)	73.9 (60.0–84.2)	92.7 (64.5–98.9)	73.9 (60.0–84.2)	92.7 (64.5–98.9)	71.3 (58.2–81.5)	92.7 (64.5–98.9)

**Table 4:** Multiple logistic regression model of each parameter for the diagnosis of FTD.

2 s/10 s						
	Wald	Coefficient	SE	OR	95% CI	Accuracy
F-theta/F-alpha	9.641	3.947	1.271	51.782	4.287–625.529	76.92%
2 s/5 s						
	Wald	Coefficient	SE	OR	95% CI	Accuracy
F-theta/F-alpha	9.636	3.943	1.270	51.594	4.278–622.182	76.92%
1 s/10 s						
	Wald	Coefficient	SE	OR	95% CI	Accuracy
F-theta/F-alpha	9.646	3.851	1.240	47.020	4.140–534.080	76.92%
2 s/10 s REST						
	Wald	Coefficient	SE	OR	95% CI	Accuracy
F-theta/F-alpha	9.439	3.500	1.139	33.115	3.551–308.830	76.92%

FTD: frontotemporal dementia; F: frontal; SE: standard error; OR: odds ratio; CI: confidence interval.

## Data Availability

The original contributions presented in the study are publicly available and can be found at the OpenNeuro Dataset, https://openneuro.org/datasets/ds004504/versions/1.0.2 (accessed on 5 December 2023).

## Supplementary Materials

The following supporting information can be downloaded at: https://www.mdpi.com/article/10.3390/medicina59122155/s1, Supplementary Figure S1. Differences in spectral power ratio, expressed as adjusted *p* value, between FTD and CTL at 2 s/5 step for FFT window length/step size per Hz. The *p* value was adjusted after FDR correction using the Benjamini–Hochberg procedure. The green and red lines represent criteria of *p* < 0.05 and *p* < 0.01, respectively; Supplementary Figure S2. Differences in spectral power ratio between FTD and CTL, expressed as adjusted *p* value, at 1 s/10 step for FFT window length/step size per Hz. The *p* value was adjusted after FDR correction using the Benjamini–Hochberg procedure. The green and red lines represent criteria of *p* < 0.05 and *p* < 0.01, respectively; Supplementary Figure S3. Differences in spectral power ratio between FTD and CTL, expressed as *p* value, with infinity reference and at 2 s/10 step for FFT window length/step size per Hz. The *p* value was adjusted after FDR correction using the Benjamini–Hochberg procedure. The green and red lines represent criteria of *p* < 0.05 and *p* < 0.01, respectively; Supplementary Figure S4. Distribution plot of each of the four spectral power ratios at 2 s/5 s with average referencing. A box-and-whisker plot is applied to present a statistical summary of each variable between the FTD and CTL groups; Supplementary Figure S5. Distribution plot of each of the four spectral power ratios at 1 s/10 s with average referencing. A box-and-whisker plot is applied to present a statistical summary of each variable between the FTD and CTL groups; Supplementary Figure S6. Distribution plot of each of the four spectral power ratios at 2 s/10 s with REST. A box-and-whisker plot is applied to present a statistical summary of each variable between the FTD and CTL groups.
