# Supplementary figures and images for: Quantitative Electroencephalography Markers for an Accurate Diagnosis of Frontotemporal Dementia: A Spectral Power Ratio Approach
Source: Medicina (Kaunas). 2023 Dec 13;59(12):2155. doi: 10.3390/medicina59122155 (PMC10744364; doi:10.3390/medicina59122155)

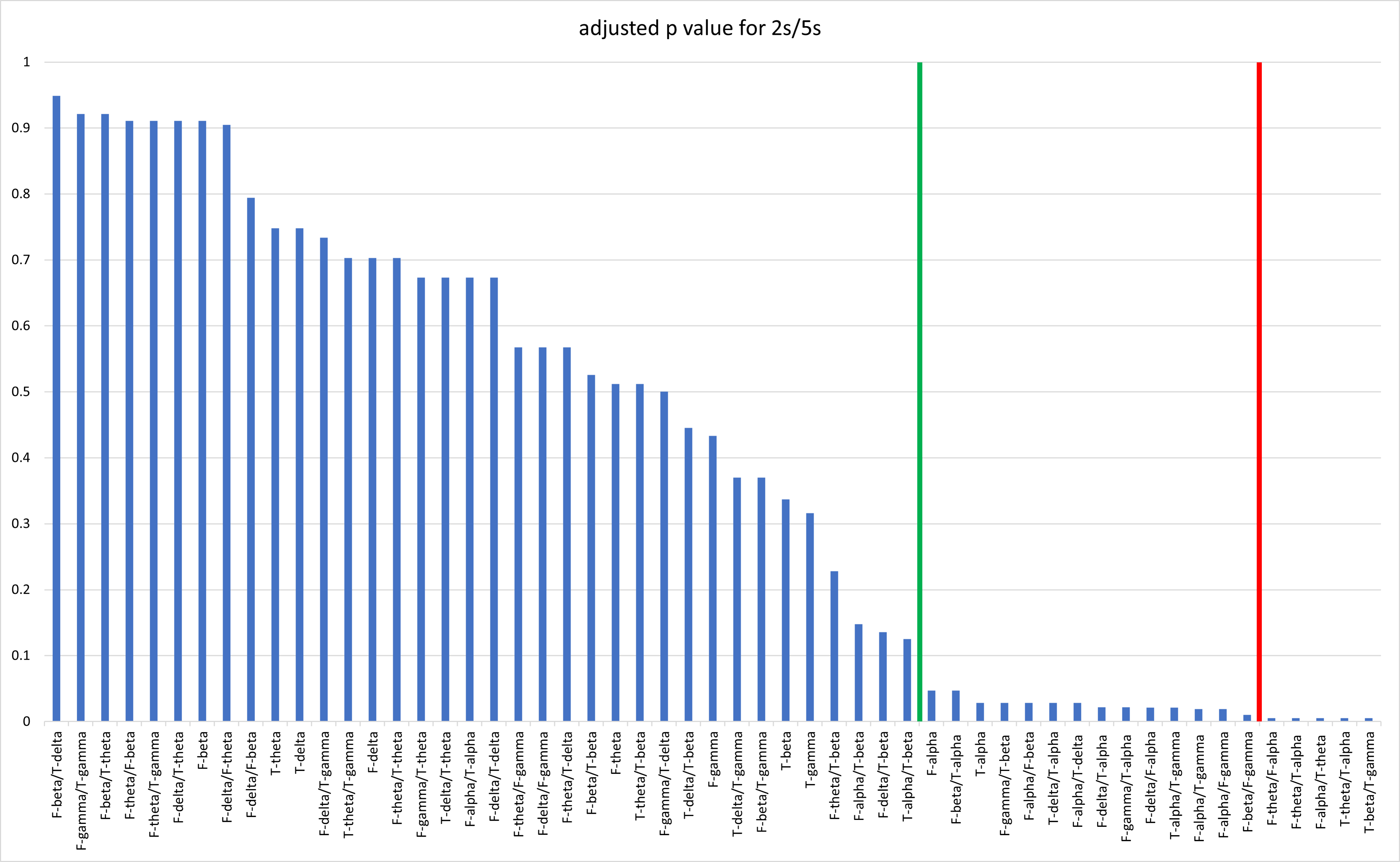

Supplement: Supplementary file 1 [file medicina-59-02155-s001.zip › Figure S1.tif]

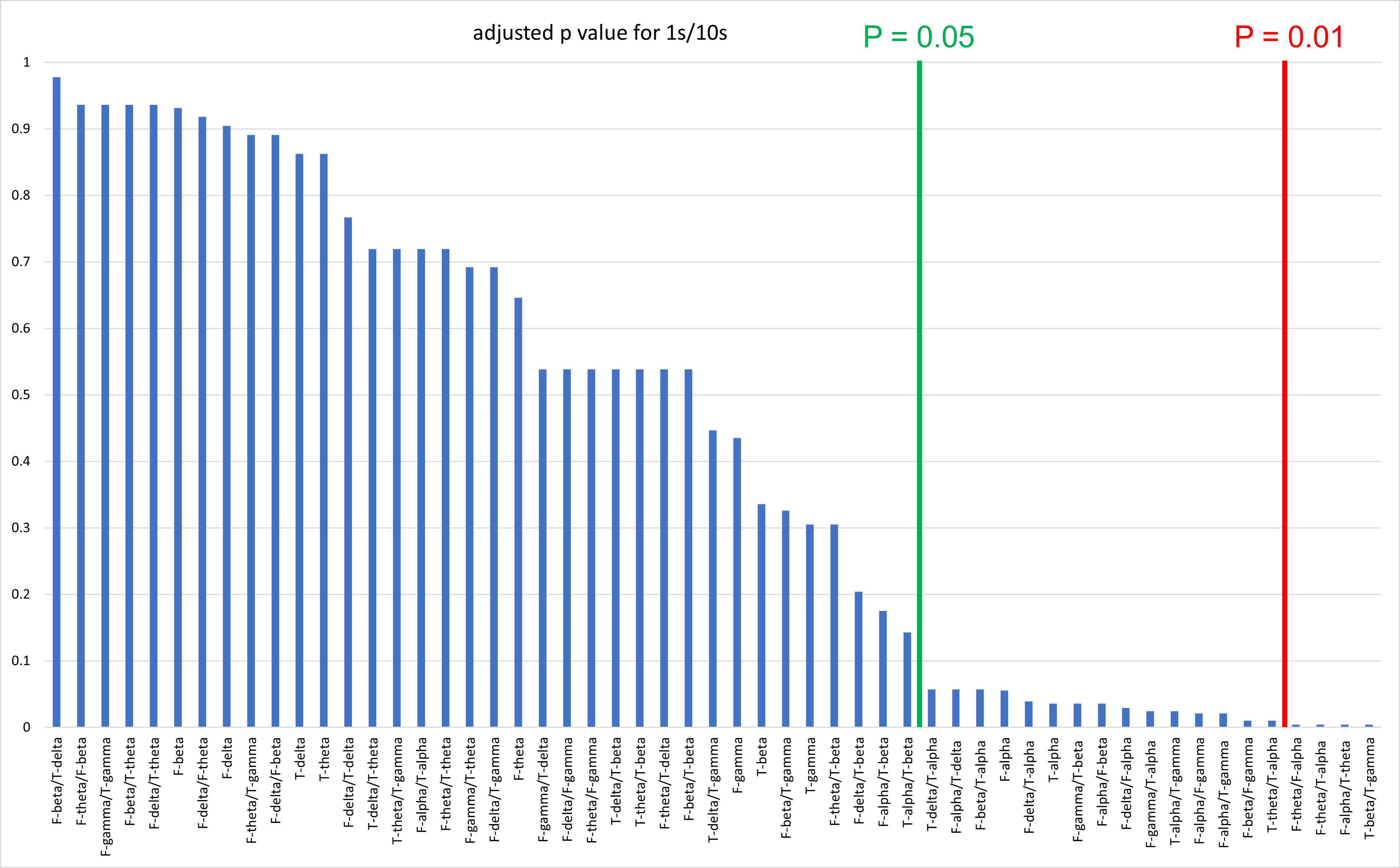

Supplement: Supplementary file 1 [file medicina-59-02155-s001.zip › Figure S2.tif]

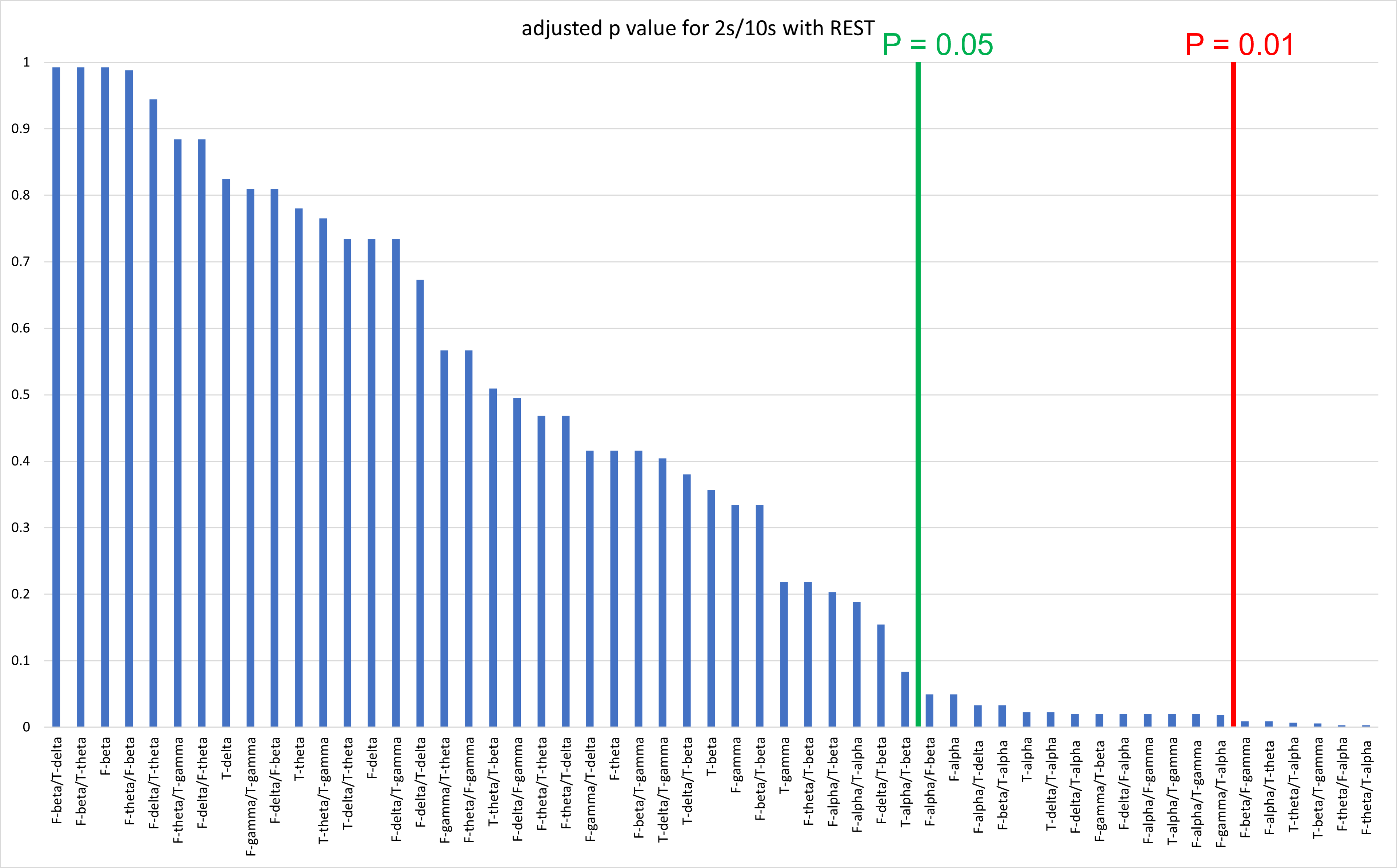

Supplement: Supplementary file 1 [file medicina-59-02155-s001.zip › Figure S3.tif]

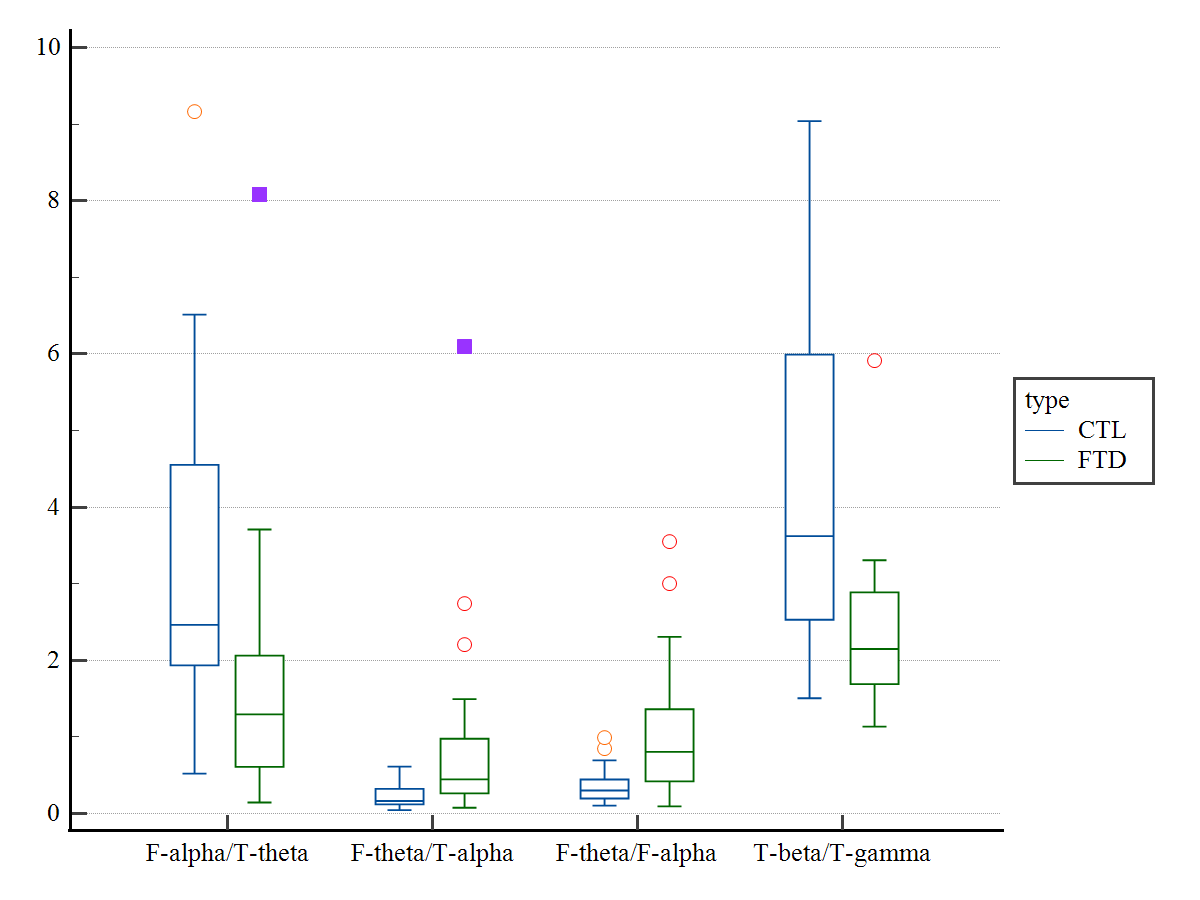

Supplement: Supplementary file 1 [file medicina-59-02155-s001.zip › Figure S4.tif]

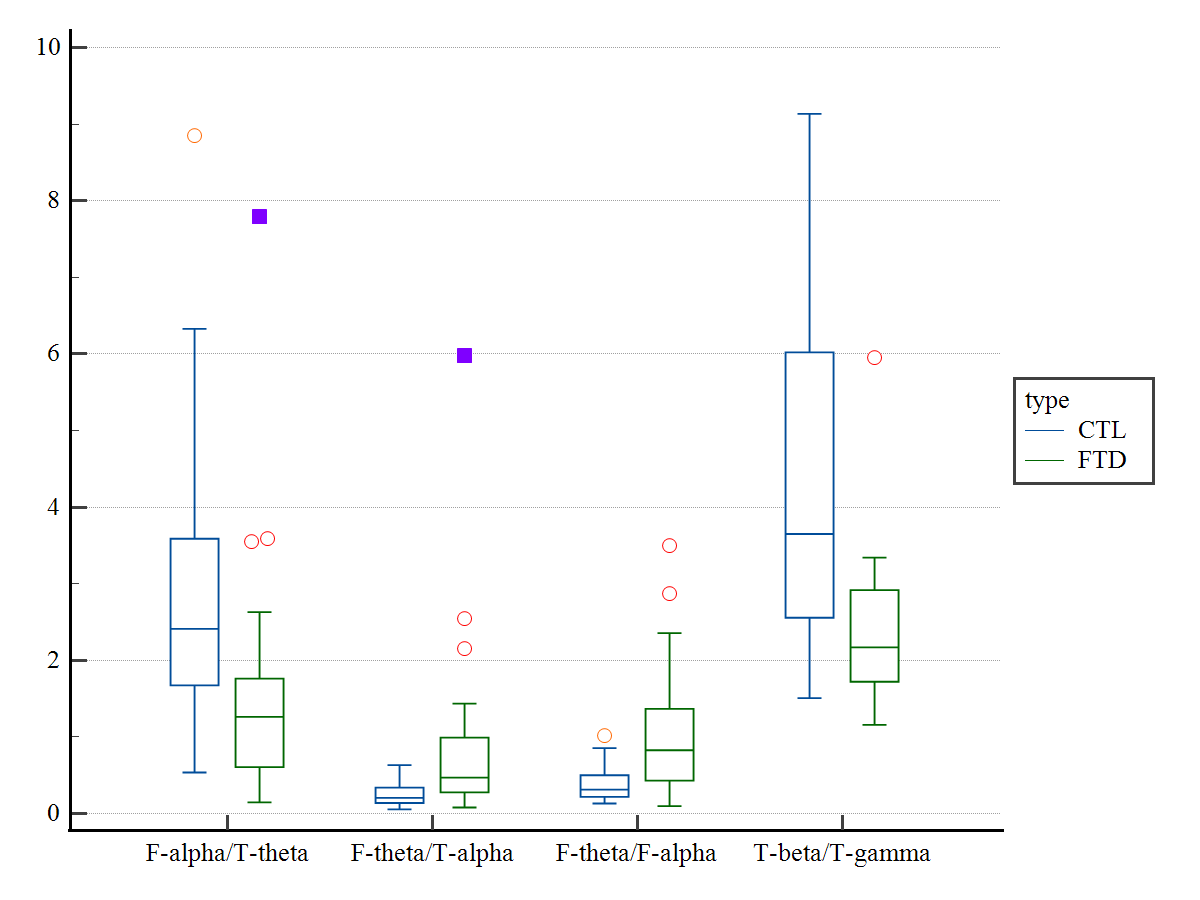

Supplement: Supplementary file 1 [file medicina-59-02155-s001.zip › Figure S5.tif]

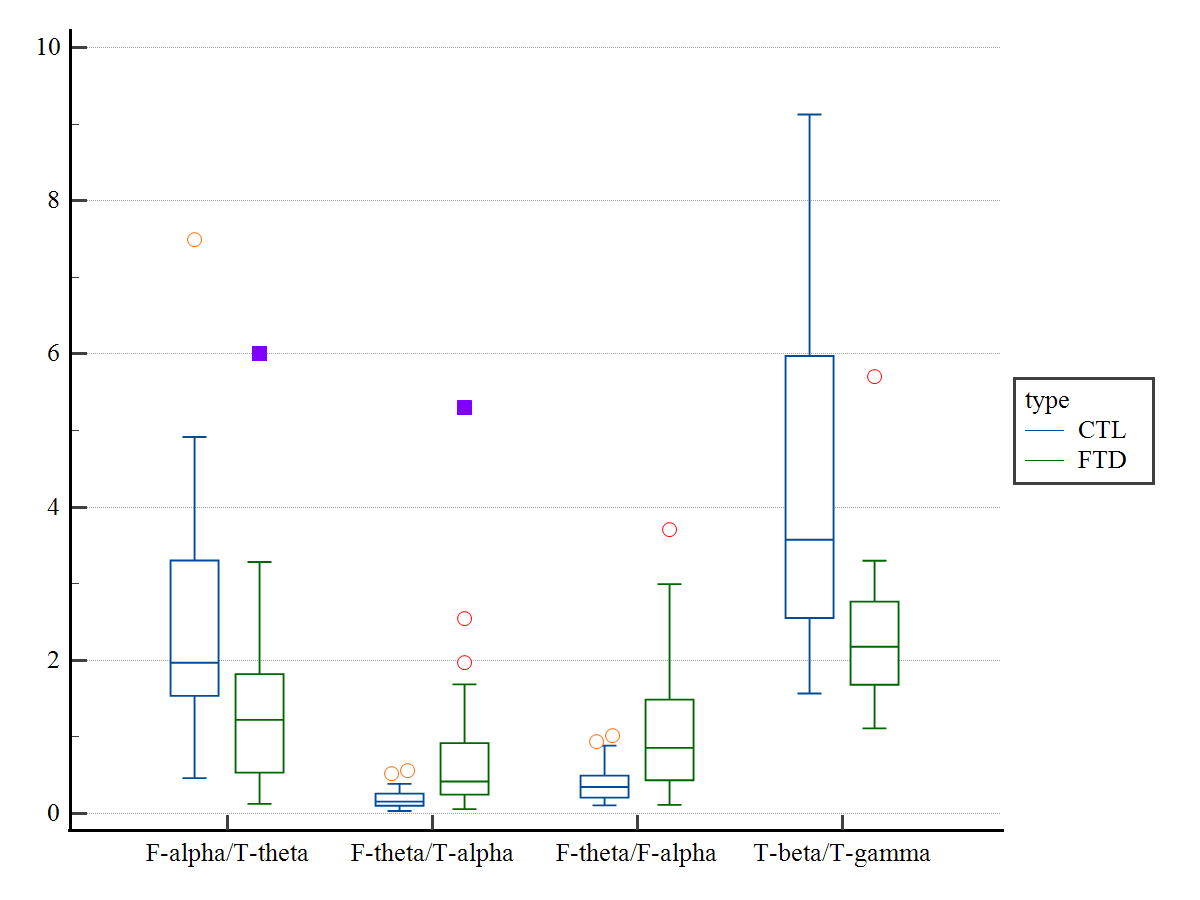

Supplement: Supplementary file 1 [file medicina-59-02155-s001.zip › Figure S6.tif]
